# Application Effects of Whole Course High-Quality Nursing on Patients with Liver Cancer during Radiotherapy

**Published:** 2019-10

**Authors:** Ling PANG, Yunxia WANG, Yuexin XING, Chunxia ZHAO

**Affiliations:** 1. Department of Radiotherapy, Liaocheng People's Hospital, Liaocheng, 252000, P.R. China; 2. Department of Hepatobiliary Surgery, Liaocheng People's Hospital, Liaocheng, 252000, P.R. China; 3. Department of Surgery, The Third Hospital of Liaocheng, Liaocheng, 252000, P.R. China

**Keywords:** Liver cancer, Radiotherapy, Depression, Nursing

## Abstract

**Background:**

To explore the application effects of whole course high-quality nursing on patients with liver cancer during radiotherapy.

**Methods:**

One hundred and five patients with liver cancer who received radiotherapy in Liaocheng People's Hospital, Liaocheng, China from March 2010 to February 2012 were selected and divided into a control group (42 cases with routine nursing) and an experimental group (63 cases with whole course high-quality nursing). The two groups of patients were observed and compared in terms of clinical data and the Activity of Daily Living (ADL) score before and after nursing (1 course of treatment/6 week). The Self-Rating Anxiety Scale (SAS) and Self-Rating Depression Scale (SDS) were used to evaluate and compare the patients' anxiety and depression before and after nursing. Adverse reactions during radiotherapy and nursing satisfactory of patients were observed and compared between the two groups.

**Results:**

After nursing, the ADL score and the nursing satisfaction in the experimental group was significantly higher than that in the control group (*P*<0.05). The SDS and SAS scores in the 2 groups before nursing were significantly higher than those after nursing (*P*<0.05). The total incidence of adverse reactions in the control group was significantly higher than that in the experimental group (*P*<0.05).

**Conclusion:**

Whole course high-quality nursing can improve the negative emotions, quality of life and nursing satisfaction, and reduce adverse reactions of patients with liver cancer during radiotherapy.

## Introduction

Liver cancer has become a common malignant tumor of the digestive system, with high incidence and mortality (1, 2). There were approximately 840,000 new cases with the incidence accounting for 4.7% of all cancers, and 780,000 deaths with the mortality accounting for 8.2% in 2018 (3). Liver cancer has unapparent symptoms in the early stage, and it has developed into in the middle and advanced stages when the symptoms occur (4). Patients with middle and advanced liver cancer cannot be operated for expected therapeutic effects. Therefore, non-surgical treatment has become the first choice for patients with liver cancer (5).

The patients who cannot be operated are currently treated by radiotherapy (6). Abbreviated as radiotherapy, radiation therapy is one of the three major treatments for cancers, and it inhibits or kills cancer cells through irradiating local malignant tumors with rays (7, 8). Patients during radiotherapy suffer from adverse reactions, such as nausea, vomiting, fever, radiodermatitis, decline of peripheral hemogram and immunosuppression. During treatment, these complications on the basis of primary diseases result in anxiety, depression, seriously decreased quality of life and even giving up treatment (9, 10). Although the routine mechanical nursing relieves patients' physical pain, it cannot meet their psychological needs. Therefore, whole course high-quality nursing based on routine nursing was carried out on the patients to solve this problem, which further reduces patients' complications during radiotherapy and helps understand their inner needs more humanely at the same time (11).

The application effects of whole course high-quality nursing on patients with liver cancer during radiotherapy were explored in this experiment, to provide references for medical staffs.

## Materials and Methods

Overall, 105 patients with liver cancer who received radiotherapy in Liaocheng People's Hospital, Liaocheng, China from March 2010 to February 2012 were selected and divided into the control group (42 cases which received routine nursing) and the experimental group (63 cases which received whole course high-quality nursing based on the control group). There were 38 males and 25 females with an average age of (51.2±8.4) years old in the experimental group, while 25 males and 17 females with an average age of (50.1±7.9) years old in the control group.

This study was approved by the Medical Ethics Committee of Liaocheng People's Hospital. Inclusion criteria: Patients who met the 7th edition of TNM staging issued by the AJCC (American Joint Committee on Cancer) in 2009 (12); patients pathologically diagnosed with primary liver cancer; patients whose first treatment was conducted in Liaocheng People's Hospital; patients who had received therapeutic regimens for liver cancer before surgery or radiotherapy; patients who signed an informed consent form.

Exclusion criteria: Patients complicated with other malignant tumors; patients with congenital cardiac and renal insufficiencies; patients who did not cooperate with treatment; patients with incomplete clinical data.

### Nursing measures

Routine nursing was performed on patients in the control group. 1) Radiotherapy knowledge was popularized to make family members cooperate with the treatment. 2) Patients' clinical data were completed. 3) Daily necessities were prepared. 4) The irradiated skin was protected from sunlight, and irritant drugs were prohibited, including soap, zinc oxide adhesive plaster, iodine tincture and alcohol. 5) Hemogram was weekly checked once to twice. 6) Education of nutrition knowledge was strengthened. 7) Hemogram, liver and kidney function, and overall physical examinations were carried out after radiotherapy. 8) The irradiated skin was protected for more than 1 month. 9. Patients were told to undergo reexamination as planned.

Whole course high-quality nursing was performed on patients in the experimental group.

### Nursing on admission

The nursing personnel warmly received the patients and their families, and carefully explained the department system, ward environment, attending doctors and primary nurses to them, so as to establish a good nurse-patient relationship. The nursing personnel patiently communicated with the patients, understood their thoughts, and evaluated their self-care ability and psychologic situation, thus making corresponding nursing plans.

### Psychological nursing

The patients changed their psychology from unacceptable to desire for survival after learning of their illness, so they hoped to stabilize their illness and prolong their life through medical treatments. They had not contacted with radiotherapy so they were fearful and depressed. Therefore, the nursing personnel appeased their emotions and explained the knowledge, precautions, postoperative complications and treatment methods of radiotherapy to them, in order to eliminate their doubts and fears. The ward environment was kept ventilated and flowers that made patients positive were placed. Cases that the disease was controlled by radiotherapy were told to patients in order to give them confidence in fighting the disease.

### Nursing during radiotherapy

1) High-quality nursing before radiotherapy. Patients were urged to form good living habits, quit smoking and drinking, go to bed early and get up early. Soft and loose cotton underwear with strong hygroscopicity was prepared, and the patients' irradiated skin was comprehensively cleaned and nursed. The importance and steps of radiotherapy were explained in detail, and the patients were escorted into the radiotherapy room in person. 2) High-quality nursing during radiotherapy. After entering the radiotherapy room, the patients were informed of the radiotherapy area, placed in a comfortable position and assisted to expose the radiotherapy site for radiotherapy. They were told not to move at will. Whether they had any adverse reactions was closely observed during radiotherapy, and unstable conditions were informed to the doctor to stop radiotherapy. 3) High-quality nursing after radiotherapy. Changes of hemogram were timely detected and recorded after radiotherapy. The attending doctors should find out the reason if the patients' white blood cells were fewer than 3.0*109/L and platelets were fewer than 8.0*109/L. Patients' irradiated skin was specially nursed and they were encouraged to undergo the next radiotherapy.

### Nursing for complications

1) Radiodermatitis. Patients' consciousness to protect the irradiated skin was strengthened, and local irradiated skin was kept dry and clean. Cleaning with alkaline soap was avoided, and irritant drugs such as alcohol, iodophor and gauze were prohibited to avoid caloric stimulation. Local erythema, pruritus and desquamation should be applied with sterilized talcum powder but not be scratched. The irradiated skin was marked and lines on the radiation fields were kept clear at all times. The doctor should be informed to redraw the lines immediately when they were fuzzy. Patients were reminded to take protective measures together with the nursing personnel, and provided with note cards to read at any time. 3% boric acid solution was compressed to keep the wound dry when there was local wet reaction. 2) Nausea, vomiting and anorexia. Whether patients had gastrointestinal reactions was closely observed. If the patients had nausea and vomiting, antiemetic drugs or needles were given to them according to the doctor's advice to relieve their pain, and then the cause of vomiting was explained. Oral cleaning and nursing was performed, and the patients' mouths were rinsed with normal saline to eliminate odors. Tea with Radix Ophiopogonis or honeysuckle was prepared for nourishing Yin and generating body fluid. 3) Fever. The patients' body temperature was monitored. Physical cooling was carried out when the body temperature was 38.5 °C, including tepid sponge bath and cold compress on the head with ice bags. Appropriate amount of warm water was prepared after sponge bath to prevent dehydration. Drug cooling was carried out when the body temperature was higher than 38.5°C, and nutrition and water were supplemented at the same time. 4) Decline of peripheral hemogram. Hemogram was weekly detected during radiotherapy because of leukopenia, and radiotherapy was immediately suspended when white blood cells were fewer than 3.0×109/L. Drugs for raising hemogram were given to the patients with obvious decline of hemogram according to the doctor's advice, and component blood transfusion or fresh whole blood transfusion was conducted when necessary. Whether there was hemorrhage was paid close attention to. The patients had significant decline of resistance, so they were easily complicated by bacterial and viral infections, which needed prevention. 5) Hepatalgia. Patients suffered from hepatalgia during treatment. The nursing personnel patiently inquired about patients' pain degree, gave them analgesics and distracted their attention. Soft music was played to relieve their pain, and books and newspapers were prepared for entertainment.

### Dietary nursing

During radiotherapy, the patients ate digestible food with high calorie, high protein, high vitamin, low fat and appropriate amount of inorganic salt, such as fish soup, custard, fruit juice, etc. Nutritional and diverse diets with perfect combination of color, aroma, taste and appearance were prepared, and the patients had many meals but little food at each. With enough vegetables and fruits eaten every day, the patients were encouraged to eat more cauliflower which could reduce cancer recurrence and metastasis. The appetite of patients was unavoidably poor due to their bland diet, so appetizing salad vegetables with less oil were prepared.

### Discharge guidance and follow-up

Discharge guidance and health education were conducted for patients. Patients' discharged registration and expense settlement were checked in, and they were encouraged to keep in touch with medical staffs using contact cards issued. Patients were instructed to take drugs whose dosage was marked with a sign pen. Patients were regularly followed up after discharge for conditions, guidance to their diets and records of the conditions of special patients.

### Observational indexes

Main observational indexes: The changes of Activity of Daily Living (ADL) score before and after nursing (1 course of treatment /6 week) were observed in the two groups of patients (13). The score had 18 test questions with a total score of 126 points and the lowest score of 18 points. The higher the score was, the stronger patients' ability of daily independent living was. The Self-Rating Anxiety Scale (SAS) and Self-Rating Depression Scale (SDS) were used to compare patients' anxiety and depression before and after nursing (14, 15). The two scores had 20 test questions altogether with a total score of 80 points and the lowest score of 20 points. The higher the score was, the more severe patients' anxiety and depression were.

Secondary observational indexes: The clinical data were compared between the two groups. Adverse reactions during radiotherapy and nursing satisfaction of patients were observed and compared between the two groups (the self-made score sheet of Liaocheng People's Hospital was used for statistics).

### Statistical analysis

SPSS 20.0 (Chicago, IL, USA) was used to analyze the data, GraphPad Prism 7 to plot figures, K-S to analyze data distribution. Count data were expressed by ratio (%) and tested by chi-square, but they were tested by chi-square with continuity correction and represented by χ^2^ when T <5 but ≥1 and n≥40. Nonparametric test was used for ranked data which were represented by Z. Measurement data were expressed by mean ± standard deviation (mean±SD), and the data conforming to normal distribution were tested by *t*. Comparison between two groups and comparison within groups before and after nursing were tested by paired t, comparison between groups tested by independent samples *t* and represented by t, the data which did not conform to normal distribution tested by rank sum and represented by Z. When *P*<0.05, there is a statistically significant difference.

## Results

### Comparison of clinical data

There was no statistically significant difference between the experimental and control groups in terms of gender, age, BMI, past medical history, place of residence, history of smoking or history of alcoholism (*P*<0.05), which indicates that the two groups of patients are comparable (Table 1).

**Table 1:** Comparison of clinical data [n(%)]

***Factors***		***Control group (n=42)***	***Experimental group (n=63)***	***χ^2^/t value***	**P *value***
Gender				0.007	0.935
	Male	25(59.52)	38(60.32)		
	Female	17(40.48)	25(39.68)		
Age (yr)		50.1±7.9	51.2±8.4	0.673	0.502
BMI (kg/m2)		22.14±1.75	21.89±1.88	0.686	0.494
Past medical history					
	Hypertension	9(21.43)	15(23.81)	0.081	0.776
	Hyperlipemia	5(11.90)	5(7.94)	0.461	0.497
	Diabetes	10(23.81)	12(19.05)	0.345	0.557
	Others	1(2.38)	1(1.59)	0.085	0.771
Place of residence				0.270	0.604
	City	28(66.67)	45(71.43)		
	Countryside	14(33.33)	18(28.57)		
History of smoking				0.837	0.425
	Yes	20(47.62)	35(55.56)		
	No	22(52.38)	28(44.44)		
History of alcoholism				0.251	0.616
	Yes	4(9.52)	8(12.70)		
	No	38(90.48)	55(87.30)		

### Changes of ADL score before and after nursing

According to the comparison, before nursing, there was no significant difference in the ADL score between the experimental and control groups (*P*>0.05). After nursing, the ADL score in the two groups was significantly improved, which in the experimental group was significantly higher than that in the control group (*P*<0.05) (Table 2).

**Table 2:** Changes of ADL score before and after nursing

***Groups***	***ADL score***		**t *value***	**P *value***
	***Before nursing***	***After nursing***		
Control group (n=42)	41.06±5.91	48.65±7.52	−7.930	<0.001
Experimental group (n=63)	41.36±6.19	63.45±5.66	−20.178	<0.001
*t*	0.248	11.492		
*P*	0.805	<0.001		

### Changes of SDS and SAS scores after nursing

Before nursing, there was no difference between the experimental and control groups in SDS or SAS scores (*P*>0.05), which were significantly higher than those after nursing (*P*<0.05). The decrease of the two scores in the experimental group was significantly greater than that in the control group (*P*<0.05) (Table 3).

**Table 3:** Changes of SDS and SAS scores after nursing

***Groups***	***SDS score***		**t/P *value***	***SAS score***		**t/P *value***
	***Before nursing***	***After nursing***		***Before nursing***	***After nursing***	
Control group (n=42)	57.48±4.27	50.17±6.43	3.861/0.001	60.23±4.11	50.83±6.94	4.426/<0.001
Experimental group (n=63)	58.11±4.52	43.23±3.86	16.130/<0.001	59.34±3.19	40.54±3.66	31.918/<0.001
*t*	0.715	6.909		1.246	9.900	
*P*	0.476	<0.001		0.216	<0.001	

### Comparison of adverse reactions during nursing

According to the comparison, there were 6 cases of leucopenia, 2 of liver function injury, 3 of gastrointestinal reactions and 2 of radiation hepatitis in the control group, while 4 of leucopenia, 3 of liver function injury, 2 of gastrointestinal reactions and 0 of radiation hepatitis in the experimental group. There was no statistically significant difference between the two groups in each adverse reaction (*P*>0.05), while the total incidence of adverse reactions in the control group was significantly higher than that in the experimental group (*P*<0.05) (Table 4).

**Table 4:** Comparison of incidence of adverse reactions [n(%)]

***Groups***	***Leucopenia***	***Liver function injury***	***Gastrointestinal reactions***	***Radiation hepatitis***	***Total***
Control group (n=42)	6(14.29)	2(4.76)	3(7.14)	2(4.76)	13(30.95)
Experimental group (n=63)	4(6.35)	3(4.76)	2(3.17)	0(0.00)	9(14.28)
χ^2^ value	1.036	0.219	0.219	1.041	4.745
*P* value	0.309	0.640	0.640	0.308	0.029

### Comparison of nursing satisfaction

According to statistics, there were 14 very satisfied patients, 22 satisfied patients and 6 dissatisfied patients in the control group, while 35 very satisfied patients, 22 satisfied patients and 6 dissatisfied patients in the experimental group. The nursing satisfaction in the experimental group was significantly higher than that in the control group (*P*<0.05) (Table 5).

**Table 5:** Comparison of nursing satisfaction [n(%)]

***Groups***	***Very satisfied***	***Satisfied***	***Dissatisfied***	**Z *value***	**P *value***
Control group (n=42)	14(33.33)	22(52.38)	6(14.29)	−2.119	0.034
Experimental group (n=63)	35(55.56)	22(34.92)	6(9.52)		

## Discussion

Liver cancer is a common malignant tumor of the digestive system, but its specific pathogenesis remains unclear (16). China had 466,100 new patients and over 420,000 deaths in 2015 (17). Therefore, the development and progression of liver cancer needs to be urgently prevented. Clinically, the disease is treated by surgery, radiotherapy and chemotherapy (18, 19). However, the patients have missed the best treatment time when admitted to hospital, so they cannot be operated. Therefore, radiotherapy and chemotherapy have become the first choice for the treatment of patients with advanced liver cancer (20).

The therapeutic effect of radiotherapy is comparable to that of surgery with the improvement of medical level, so radiotherapy is one of the best schemes for patients who cannot be operated or whose surgical risks outweigh the benefits (21). However, patients with liver cancer are prone to leukopenia, liver function injury and other adverse reactions during treatment, which easily results in negative emotions and decreased quality of life. This makes patients question the effectiveness of treatment and greatly reduces the therapeutic effect and compliance. Therefore, nursing is usually performed to adjust patients' negative emotions and improve their quality of life.

Clinical nursing plans are routine nursing, which cannot meet patients' needs with the improvement of living standards and quality of life. Therefore, it is particularly important to explore a new nursing plan to improve patients' negative emotions and quality of life. Compared with routine nursing, effective and patient-centered whole course high-quality nursing comprehensively and objectively evaluates patients' conditions, so as to formulate reasonable nursing plans. It provides a comfortable treatment environment for patients, strengthens the doctor-patient communication during each nursing link, and respects the service concept, thus establishing a good doctor-patient relationship (22). There is no previous study showing the effects of whole course high-quality nursing on the adverse reactions and quality of life of patients with liver cancer during radiotherapy, which was therefore explored in this study to provide references for medical staffs. The ADL score, which was used to evaluate the patients' quality of life in this study, is a scale for evaluating patients' ability of daily living, and markedly effective in evaluating the quality of life of patients with diseases (23). In the experimental and control groups in this study, the ADL score after nursing was significantly better than that before nursing, and the score in the experimental group was significantly higher than that in the control group during nursing. The quality of life of patients with liver cancer is improved by humanistic care (24). Although their nursing plan is different from ours, the patients' quality of life was improved by whole course high-quality nursing in this study, which indicates that whole course high-quality nursing can improve the quality of life. This is mainly because appropriate nursing plans before, during, and after nursing reduce patients' pressure.

SDS and SAS scores were compared between the two groups in this study. SDS and SAS are commonly used and markedly effective scales for evaluating patients' anxiety and depression in nursing plans (25). The results of this study showed that after nursing, the two scores in the two groups were significantly decreased, and the scores in the experimental group were lower than those in the control group. The SAS and SDS scores of patients with liver cancer during radiotherapy are significantly improved by psychological nursing (26). Psychological nursing for patients' emotions was carried out during whole course high-quality nursing in this study, and patients' anxiety and depression were improved. This indicates the effectiveness of whole course high-quality nursing in improving patients' negative emotions. Prevention and nursing plans for complications were also carried out during whole course high-quality nursing when compared with psychological nursing. In this study, there was no difference between the two groups in each adverse reaction, while the total incidence of adverse reactions in the control group was significantly higher than that in the experimental group. It is indicated that whole course high-quality nursing can reduce and prevent adverse reactions. Finally, the nursing satisfaction in the experimental group was significantly higher than that in the control group. The nursing plan for patients in the experimental group was follow-up. Patients' questions were answered in time, and the patients were guided and followed up after discharge, so their satisfaction was improved.

This study confirms the effects of whole course high-quality nursing on patients with liver cancer during radiotherapy, but it still has limitations. Firstly, there were a lot of nursing personnel in this study, which inevitably wasted limited resources. Secondly, the patients were only followed up for a short time due to the short research time. Therefore, in subsequent studies, the follow-up time should be prolonged and the long-term effects of whole course high-quality nursing on the patients' quality of life and adverse reactions should be observed, in order to prove the effectiveness of this study and reduce medical staffs and unnecessary expenses.

## Conclusion

Whole course high-quality nursing can improve the negative emotions, quality of life and nursing satisfaction, and reduce adverse reactions of patients with liver cancer during radiotherapy.

## Ethical considerations

Ethical issues (Including plagiarism, informed consent, misconduct, data fabrication and/or falsification, double publication and/or submission, redundancy, etc.) have been completely observed by the authors.
